# Efficacy and Safety of Anti-CD38 Antibody-Containing Triplet Regimens in Frail Patients with Multiple Myeloma

**DOI:** 10.3390/cancers18071048

**Published:** 2026-03-24

**Authors:** Hirono Iriuchishima, Akio Saito, Masahiro Mihara, Shuhei Kanaya, Ryo Yoshizawa, Atsushi Isoda, Morio Matsumoto

**Affiliations:** 1 Department of Clinical Research, NHO Shibukawa Medical Center, Shibukawa 377-0280, Gunma, Japan; 2 Department of Hematology, NHO Shibukawa Medical Center, Shibukawa 377-0280, Gunma, Japan; saito.akio.hf@mail.hosp.go.jp (A.S.); mihara.masahiro.ks@mail.hosp.go.jp (M.M.); kanaya.shuhei.tk@mail.hosp.go.jp (S.K.); hoshiclinic01@gmail.com (A.I.); matsumoto.morio.zr@mail.hosp.go.jp (M.M.); 3 Department of Hematology, Hoshi Clinic, Maebashi 379-2131, Gunma, Japan

**Keywords:** multiple myeloma, anti-CD38 antibody-containing triplet regimen, frail patient

## Abstract

**Background/Objectives**: Although triplet regimens have been shown to be effective and safe in pivotal studies involving frail patients with multiple myeloma (MM), their use in frail patients is often avoided in real-world settings. As MM treatment progresses and options increase, it is crucial to clarify the efficacy and safety of triplet regimens in clinical practice. **Methods**: Patients who received anti-CD38 antibody-containing triplet regimens at our hospital from 2017 to 2024 were divided into frail and non-frail groups based on the IMWG simplified frailty scale and retrospectively analyzed. **Results**: In the 150 patients included, the median age was 76 years for the frail group and 69 years for the non-frail group. Daratumumab-containing triplet regimens were given to 108 (82 frail) patients, and isatuximab-containing triplet regimens were given to 42 patients (18 frail). Progression-free survival and overall survival for the frail and non-frail groups were 15.4 vs. 11.4 months and 45.6 vs. 40.7 months, respectively; the overall response rate was 76% vs. 68%, with no significant differences. Prognoses by regimen were also not significantly different. There were no significant differences in any adverse events and grade 3–4 hematological and non-hematological adverse events between the two groups. This analysis showed that, in frail MM patients who were eligible to receive triplet regimens, anti-CD38 antibody-containing triplet regimens were as effective and safe as in non-frail patients. **Conclusions**: In conclusion, these regimens may be viable options for frail patients, provided that appropriate management, including withdrawal of therapeutic agents, dose reduction, and infection control, is rigorously performed, as for non-frail patients.

## Simple Summary

In recent years, the treatment options for patients with multiple myeloma have advanced significantly with the arrival of quadruplet combination therapy, chimeric antigen receptor T-cell therapy, and bispecific antibody drugs. However, treatment options for the majority of patients—elderly and/or frail patients—remain debated. Evaluating the efficacy and safety of triplet regimens, including anti-CD38 antibodies, the conventional standard therapy for transplant-ineligible MM patients, is critical for determining future treatment strategies for these patients. Therefore, a retrospective analysis of this issue was performed by dividing patients into frail and non-frail categories. It was found that anti-CD38 antibody-containing regimens in frail patients with multiple myeloma who were eligible to receive triplet regimens demonstrated equivalent efficacy and safety to those observed in non-frail patients, when patients in both groups were managed similarly (including initial dose, infection prevention, and appropriate dose reduction during treatment).

## 1. Introduction

Multiple myeloma (MM) is one of the most common neoplastic diseases of older adults, with a median age at diagnosis of approximately 70 years, and over 60% of diagnosed patients are over 65 years of age [1]. According to the systematic review evaluating the prevalence of frailty, the proportion of frail patients with MM has been reported to range from approximately 17.2% to 73.6% [2]. In MM, frailty is associated with an increased risk of mortality, disease progression, adverse events, and treatment discontinuation [3]. Several methods for assessing frailty have been proposed, with IMWG (age, comorbidities, and patient-evaluated self-care and household management assessments using the Katz Activity of Daily Living) [3], R-MCI (age, Karnofsky performance status, renal function, and high-risk chromosomal abnormalities) [4], and Mayo risk score (age, N-terminal pro-brain natriuretic peptide, Eastern Cooperative Oncology Group (ECOG)) [5] being commonly used. However, they are somewhat complicated in actual clinical practice. The simplified frailty scale (age, Charlson Comorbidity Index, and ECOG performance status) was later proposed by Facon et al. [6] and has been evaluated as sufficiently valuable for assessing frail and non-frail individuals. It is now commonly used in various clinical trials.

Due to the use of novel first-generation agents, including bortezomib, a proteasome inhibitor, and lenalidomide, a second-generation immunomodulatory agent, progression-free survival (PFS) and overall survival (OS) have shown significant improvements [7,8,9,10]. These benefits have also been demonstrated in elderly patients [11]. However, the approval of the anti-CD38 monoclonal antibodies daratumumab and isatuximab has further significantly improved the prognosis for patients with MM, marking a stark contrast to previous outcomes [12,13,14,15,16,17,18,19,20,21,22,23]. Consequently, this triplet combination therapy using anti-CD38 monoclonal antibodies has become the mainstream treatment worldwide for both newly diagnosed MM (NDMM) and relapsed/refractory MM (RRMM). In MM with a high proportion of elderly patients, the triplet regimen, including anti-CD38 monoclonal antibody therapy, has demonstrated sufficient efficacy and safety, even in frail patients who meet the eligibility criteria of the pivotal study [24,25,26].

In recent years, MM treatments have expanded with the arrival of quadruplet regimens [27,28], chimeric antigen receptor (CAR)-T therapy [29], and bispecific antibody drugs [30,31,32]. In this context, clarifying the efficacy and safety of triplet regimens incorporating anti-CD38 monoclonal antibody drugs in frail patients in real-world clinical practice is considered crucial for future treatment selection. The triplet regimen containing anti-CD38 monoclonal antibodies is widely used even for frail patients, though real-world clinical data remain insufficient. The objective of this study was to clarify future treatment options by evaluating the real-world efficacy and safety of a triplet regimen containing anti-CD38 monoclonal antibody drugs in frail MM patients.

## 2. Materials and Methods

### 2.1. Patients

We conducted a single-center retrospective cohort study of patients with MM who were treated with anti-CD38 monoclonal antibody-containing triplet regimens between October 2017 and December 2024 at Shibukawa Medical Center. The regimen for each patient was determined at weekly conferences held by hematologists. The patients were divided into a frail group and a non-frail group. Frailty was assessed using the simplified frailty scale, combining the patient’s age, comorbidities (Charlson Comorbidity Index-CCI), and ECOG performance status (PS) [6]. The frail group was defined as having a score of ≥2, with the non-frail group having a score of 0–1. Data on the clinical features of patients at diagnosis, including age, sex, M-protein, treatment regimens, and clinical stages according to the revised international staging system (R-ISS), were collected from their electronic medical records. Fluorescence in situ hybridization (FISH) data at the time of initial diagnosis were assessed for all NDMM patients. Other FISH data, including t(4;14)(FGFR3 and IGH), del(17p)(TP53), and t(14;16)(IGH and MAF), which are defined as high-risk cytogenetic profiles, were also examined [33]. Response rates, PFS, OS, relative dose intensity (RDI), adverse events (AEs), G-CSF use, prophylactic medication, and the implementation of immunoglobulin replacement therapy (IgRT) were analyzed for all patients. RDI is calculated based on the initial dose administered at the time of induction, as determined by the attending physician. AEs were evaluated based on Common Terminology Criteria for Adverse Events v 5.0 (CTCAE v5.0), and responses were assessed according to the International Myeloma Working Group (IMWG) response criteria. The local institutional review board approved this study.

### 2.2. Statistical Analysis

Univariate analyses between dichotomous variables were performed using Fisher’s exact test or the Mann–Whitney U test. Survival curves were constructed according to the Kaplan–Meier method, and differences were compared using the log-rank test. In all statistical tests, a *p*-value < 0.05 was considered significant, and statistical analysis was performed using EZR version 1.52.

## 3. Results

### 3.1. Baseline Features (Table 1)

A total of 150 patients were included, with 47 having NDMM and 103 having RRMM. The median age of all patients was 71 [42–88] years; the frail group was significantly older than the non-frail group, with a median age of 76 [49–88] years compared with 69 [42–79] years (*p* < 0.001); and 84 (56%) patients were male. Although all non-frail patients had ECOG PS 0-1, 23 (32%) patients with ECOG PS 2 and 14 (20%) patients with PS 3 (*p* < 0.001) were frail. The major immunophenotype of all patients included IgG (55%), IgA (20%), and Bence Jones protein (BJP) (18%), with no significant difference between the two groups. The proportion of patients with NDMM tended to be higher in the frail group (39% vs. 24%, *p* = 0.05). The majority of patients had R-ISS stage II (55%) or stage III (29%) with no significant difference between the two groups (*p* = 0.51). Renal function was assessed using the estimated glomerular filtration rate (eGFR), which was less than 60 mL/min/1.73 m^2^ in 87 (58%) patients and was comparable between the two groups (*p* = 0.62). There were significantly more patients with high-risk chromosomal abnormalities in the non-frail group (59% vs. 72%, *p* = 0.03). The anti-CD38 monoclonal antibody-containing triplet regimens were daratumumab, lenalidomide, and dexamethasone (DRd) (44%); isatuximab, pomalidomide, and dexamethasone (IsaPd) (20%); isatuximab, carfilzomib, and dexamethasone (IsaKd) (8%); daratumumab, bortezomib, and dexamethasone (DBd) (21%); daratumumab, carfilzomib, and dexamethasone (DKd) (3%); and daratumumab, pomalidomide, and dexamethasone (DPd) (5%). No significant difference in regimen selection was observed between the two groups, but carfilzomib-containing triplet regimens tended to be avoided in frail patients (25% vs. 75%, *p* = 0.077).

**Table 1:** Baseline clinical characteristics of 150 patients.

	Frail (*n* = 71)	Non-Frail (*n* = 79)	All (*n* = 150)	*p*-Value
Age, year, median (range)	76 (49–88)	69 (42–79)	71 (42–88)	<0.001
Sex, male/female (*n*)	41/30	43/36	84/66	0.74
ECOG PS, *n* (%)				<0.001
0–1	34 (48)	79 (100)	113 (75)	
2	23 (32)	0 (0)	23 (15)	
3–4	14 (20)	0 (0)	14 (9)	
Paraprotein (isotype)				0.90
IgG, *n* (%)	41 (58)	41 (52)	82 (55)	
IgA, *n* (%)	14 (20)	16 (20)	30 (20)	
BJP, *n* (%)	13 (18)	14 (18)	27 (18)	
NDMM, *n* (%)	28 (39)	19 (24)	47 (31)	0.05
R-ISS stage				0.51
I, *n* (%)	7 (10)	8 (10)	15 (10)	
II, *n* (%)	37 (52)	45 (57)	82 (55)	
III, *n* (%)	24 (34)	19 (24)	43 (29)	
eGFR < 60 mL/min/1.73 m^2^, *n* (%)	43 (61)	44 (56)	87 (58)	0.62
High-risk cytogenetics	42 (59)	57 (72)	99 (66)	0.03
Regimen				0.12
DRd	38 (54)	28 (35)	66 (44)	
IsaPd	10 (14)	20 (25)	30 (20)	
IsaKd	3 (4)	9 (11)	12 (8)	
DBd	15 (21)	16 (20)	31 (21)	
DKd	1 (1)	3 (4)	4 (3)	
DPd	4 (6)	3 (4)	7 (5)	

ECOG PS = Eastern Cooperative Oncology Group performance status; NDMM = newly diagnosed multiple myeloma; R-ISS = revised international staging system; eGFR = estimated glomerular filtration rate; DRd = daratumumab, lenalidomide, and dexamethasone; IsaPd = isatuximab, pomalidomide, and dexamethasone; IsaKd = isatuximab, carfilzomib, and dexamethasone; DBd = daratumumab, bortezomib, and dexamethasone; DKd = daratumumab, carfilzomib, and dexamethasone; DPd = daratumumab, pomalidomide, and dexamethasone.

### 3.2. The Relative Dose Intensity of Treatment

The median RDIs of the anti-CD38 antibodies daratumumab and isatuximab were 100% and 96%, respectively, in all patients, and there were no significant differences between the frail and non-frail groups (*p* = 0.21). The median RDI of bortezomib (92% vs. 100%) and dexamethasone (87% vs. 97%) tended to be lower in the frail group than in the non-frail group (*p* = 0.06 and 0.09, respectively), whereas that for other therapeutic agents was equivalent in both groups (Table 2).

There were no differences between the frail and non-frail groups in the proportion of patients who had their lenalidomide (41% vs. 50%, respectively, *p* = 0.62), pomalidomide (43% vs. 58%, respectively, *p* = 0.5), and dexamethasone (30% vs. 19%, respectively, *p* = 0.18) doses reduced during the course of treatment.

Comparing initial doses at treatment initiation, lenalidomide showed a median dose of 15 mg in frail patients and 10 mg in non-frail patients, with no difference in the proportion requiring dose reduction from baseline (55% vs. 68%, *p* = 0.32). However, for pomalidomide, the median initial dose was 3.5 mg in frail patients and 4 mg in non-frail patients. The proportion requiring dose reduction from baseline was 50% in frail patients and 8.7% in non-frail patients, indicating a significantly higher proportion in frail patients (*p* = 0.014) (Table 3).

In such situations, when treatment was initiated, the proportion of patients who interrupted or discontinued lenalidomide (56% vs. 54%, respectively, *p* = 1.0) or dexamethasone (7% vs. 10%, respectively, *p* = 0.57) during treatment showed no difference between the frail and non-frail groups. Furthermore, the proportion of patients who discontinued the entire regimen tended to be slightly higher in the non-frail group (69% vs. 82%, respectively, *p* = 0.08). Regarding reasons for discontinuation of treatment, disease progression and lack of efficacy were the most common in both groups (51% vs. 62%, respectively). Discontinuation due to AEs was observed in 8.5% of frail patients and 16% of non-frail patients, and there was no significant difference in AE-related discontinuation rates between these groups (*p* = 0.45, Table 4).

### 3.3. Efficacy

**Figure 1:**
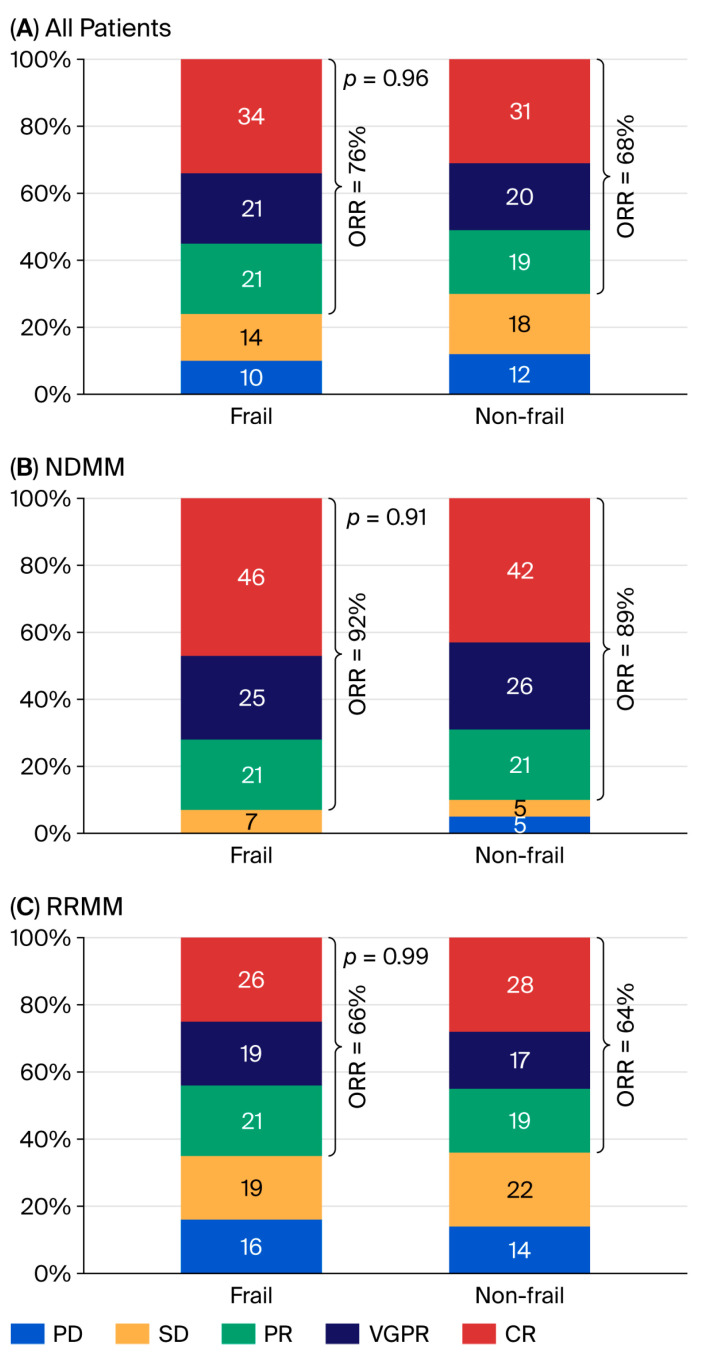
Best response to treatment by frailty. CR = complete response, VGPR = very good partial response, PR = partial response, SD = stable disease, PD = progressive disease, ORR = overall response rate.

The overall response rate (ORR) was 76% in the frail group and 68% in the non-frail group, among which 24 (34%) patients achieved a complete response (CR) and 15 (21%) achieved a very good partial response (VGPR) in the frail group. By contrast, 24 (31%) patients achieved CR and 15 (20%) achieved VGPR in the non-frail group. The efficacy was nearly equivalent in the two groups (*p* = 0.96) (Figure 1A). Next, we analyzed both NDMM and RRMM patients, and similarly, no significant difference was observed in ORR between both groups (Figure 1B,C).

**Figure 2:**
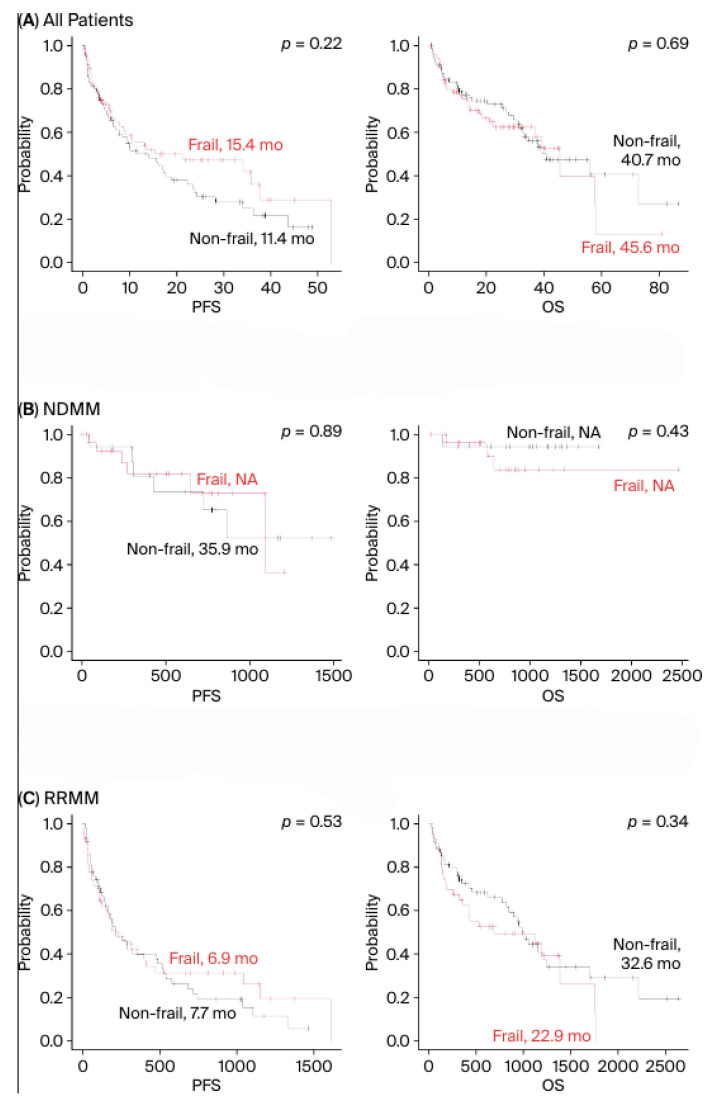
Progression-free survival and overall survival by frailty. PFS = progression-free survival; OS = overall survival.

The median follow-up for the entire cohort was 680 (26–2635) days. The estimated median PFS was 15.4 months in the frail group and 11.4 months in the non-frail group, with no significant difference (*p* = 0.22), whereas the median OS was 45.6 months in the frail group and 40.7 months in the non-frail group, with no significant difference (*p* = 0.61) (Figure 2A). Next, we analyzed the median PFS divided into NDMM and RRMM patients, and similarly, no significant difference was observed in PFS between both groups (Figure 2B,C).

**Figure 3:**
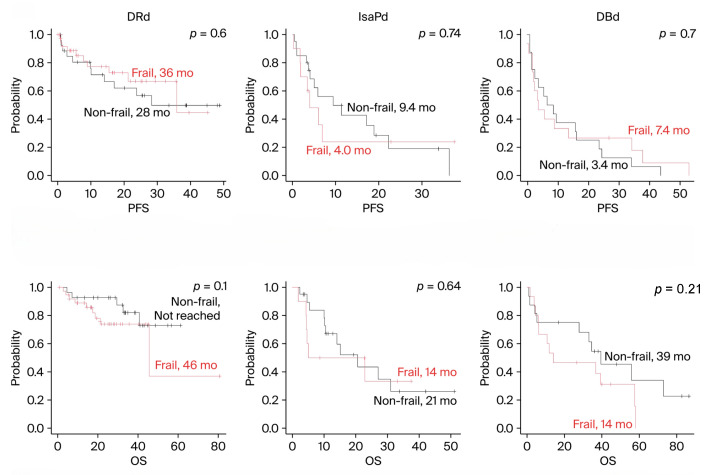
Progression-free survival and overall survival by regimen. PFS = progression-free survival; OS = overall survival; DRd = daratumumab, lenalid omide, and dexamethasone; IsaPd = isatuximab, pomalidomide, and dexamethasone; DBd = daratumumab, bortezomib, and dexamethasone.

**Figure 4:**
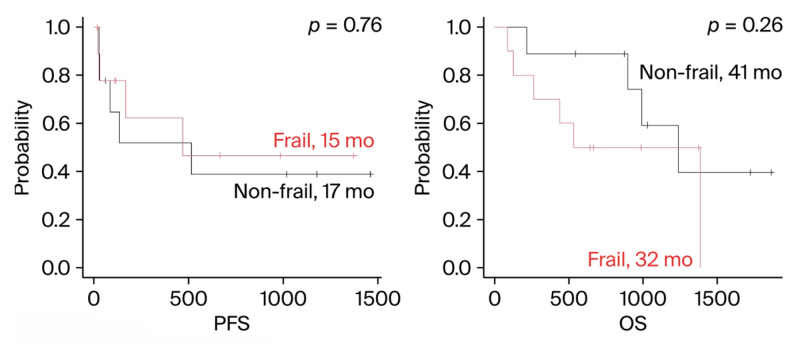
Progression-free survival and overall survival in DRd for RRMM. PFS = progression-free survival; OS = overall survival.

Next, PFS and OS were compared by treatment regimen. When examining the most common regimens, DRd, IsaPd, and DBd, no difference in prognosis was observed between frail and non-frail patients across any regimen (Figure 3). Both IsaPd and DBd were obtained from RRMM patients. Since the DRd shown in Figure 3 included both NDMM and RRMM patients, we reanalyzed the data specifically for RRMM patients, but the results remained consistent (Figure 4). The results of the DRd in NDMM were the same as those in Figure 2B.

### 3.4. Safety

A total of 134 (89.3%) patients reported G3-4 AEs. As shown in Table 5, the most frequent hematological AEs were lymphocytopenia (*n* = 108, 72%), neutropenia (*n* = 87, 58%), and anemia (*n* = 49, 33%). However, there were no significant differences between the frail and non-frail groups in hematological AEs.

The most frequent G3-4 non-hematological AEs included infection (*n* = 21, 14%), pneumonia (*n* = 16, 11%), and sepsis (*n* = 4, 2.7%). There were also no differences in non-hematological toxicity incidence rates between the two groups. One non-frail patient died from septic shock caused by ESBL-producing Escherichia coli during the third course of DBd therapy.

There was no significant difference between the frail and non-frail groups in the frequency of G-CSF use (46.5% vs. 43%, *p* = 0.74) or prophylactic medication (one at 39.4% and two at 50.7% vs. one at 32.9% and two at 62%, respectively; *p* = 0.37). On the other hand, IgRT tended to be more common in the frail group (23.9% vs. 16.5%, *p* = 0.31).

## 4. Discussion

In this study, we analyzed real-world data characterizing frail MM patients who were treated with triplet regimens containing anti-CD38 monoclonal antibody drugs. There have been sub-analyses of pivotal studies on frailty in triplet combination therapy using anti-CD38 antibody drugs. In the subgroup analysis of the phase 3 MAIA study of transplant-ineligible NDMM by frailty status, PFS was not reached in frail patients receiving DRd, with a good MRD-negative rate of 23.7%. However, the incidence of grade 3–4 AEs, including neutropenia (57.7% vs. 44.1% for frail vs. non-frail, respectively), pneumonia (19.6% vs. 10.3% for frail vs. non-frail, respectively), and death (11.9% vs. 1.5% for frail vs. non-frail, respectively), was higher in the frail group than in the non-frail group. Furthermore, the treatment duration was shorter in the frail group at 25.8 months compared with 33.6 months in the non-frail group, and the RDI of lenalidomide in the frail group was 65.4%, lower than the 77.2% in the non-frail group [24]. By contrast, analysis of NDMM patients treated with DRd therapy in the present cohort showed no significant difference in treatment duration between the frail group (15.5 months) and the non-frail group (14.8 months) (*p* = 0.40). Another anti-CD38 antibody drug, isatuximab, has also demonstrated high tolerability. In the subgroup analysis, PFS in the frail group was 9 months compared to 12.7 months in the non-frail group. The rate of VGPR or better was also good, at 29.2% vs. 34.7% for frail vs. non-frail patients, respectively. Nevertheless, grade 4 neutropenia was more common in the frail group (68.8% vs. 58.0% for frail vs. non-frail, respectively). Other severe AEs and treatment durations were reported to be comparable between the two groups (91.7% vs. 86% and 40.8 vs. 41.6 weeks, respectively) [26].

In the present analysis, the choice of treatment with the anti-CD38 antibody-containing triplet regimen was equivalent in both groups. While statistically equivalent, carfilzomib tends to be avoided in the frail group, suggesting a physician bias in treatment selection for frail patients. However, even though more than half of the frail group had a PS score ≥ 2, the frail group demonstrated equivalent RDI, efficacy, and safety to the non-frail group. The discontinuation rate was higher in the non-frail group, with disease progression identified as the main reason. This suggests a possible association with the significantly higher proportion of HRCA in the non-frail group.

To date, real-world data have shown that doublet regimens were more frequently selected for elderly patients [34,35,36]. Gonzalez-Lugo et al. analyzed patients diagnosed with MM at ≥70 years of age between 2000 and 2017. Although this analysis predated the introduction of anti-CD38 antibody drugs, in the majority of cases, doublet regimens were used over triplet regimens (68% vs. 32%) and treatment changes were more common in doublet regimens compared with triplet regimens (71% vs. 42.4%, *p* < 0.01). By contrast, the occurrence of a PR or better did not differ between doublets and triplets (61.7% vs. 66.7%), and there was no difference in the frequency of AEs among groups [34]. In other words, this report indicates that triplet regimens do not necessarily yield superior therapeutic effects.

However, in recent years, the trend seems to be shifting. Tyczyńska et al. analyzed MM in patients over 75 years of age between 2018 and 2019. They reported that 79.9% of the frail group (IMWG frailty score) received triplet regimens (not including anti-CD38 antibodies), not only in reduced doses. Surprisingly, 77.8% of ECOG 3 and 87.5% of ECOG 4 patients were selected for triplet regimens. Unfortunately, though the triplet regimens demonstrated superior ORR, patients with an ECOG ≥ 3 achieved VGPR or better in only a few cases [37].

In a recent prospective study (MFRAIL) of patients aged 75 years or older, Haider et al. reported that 84.5% of patients were administered triplet regimens, including anti-CD38 antibodies, and there were no significant differences in the selection of triplet or doublet therapy between frail and non-frail patients. They found only an association between frailty and non-steroid MM drug reduction [38]. By contrast, DuMontier et al. reported that MM patients with moderate to severe frailty showed the strongest association with reduced mortality in VRd compared with Rd [39], suggesting that triplet regimens should not be avoided in frail patients.

In the most recent report, Shpitzer et al. analyzed treatment approaches and prognosis in 652 patients aged ≥ 70 years. Multivariate analysis showed that triplet/quadruplet combination therapy was associated with improved time to next treatment (TTNT) and OS compared with doublet therapy. They also reported that daratumumab-based regimens prolonged TTNT across all age groups. Furthermore, they reported that a high CCI score and age ≥ 80 years were predictors of poor OS [40].

As myeloma treatment advances and diversifies, it is essential to accurately assess the “truly frail”. Though its definition is extremely difficult and ambiguous, it is important that the attending physician and multiple doctors conduct a holistic assessment that encompasses the patient’s social background, one that existing scoring systems cannot evaluate.

The present data demonstrated very good efficacy and safety even in frail patients. Although no significant difference was observed, G3-4 infections and pneumonia tended to be more frequent in the frail group. Regarding infection prevention, the frequency of G-CSF use and the content of prophylactic medications were comparable between the frail and non-frail groups, but IgRT was more commonly administered in the frail group. The anti-CD38 antibody-containing triplet regimens in frail patients did not exhibit a high rate of discontinuation due to AEs when initial dose determination, treatment interruption or dose reduction, and infection control management were appropriately performed as in non-frail patients.

On the other hand, it is known that the number of naive T cells in the elderly decreases with aging due to thymic atrophy [41,42], and CD38 is also expressed on T cell subsets. It has been reported that anti-CD38 antibody drugs can deplete CD38-positive regulatory immune cells, reduce the proportion of naive T cells, and increase the proportion of memory T cells [43]. Therefore, regardless of frailty status, elderly patients require careful management for various infections, including opportunistic infections. Globally, the average lifespan is increasing, and we are no longer in an era where pharmacotherapy must be avoided simply because a patient is elderly. Nowadays, in addition to conventional anticancer drugs (cytotoxic anticancer drugs), new types of anticancer drugs (drugs called “molecular targeted therapies”, “immunotherapy, including bispecific antibodies, CAR-T therapy”, and “immune checkpoint inhibitors”) are used, greatly expanding the range of pharmacotherapies for cancers, including MM, compared to before. Therefore, it is considered crucial to select the appropriate treatment from among these diverse options, even for frail patients.

This study has limitations. This was a retrospective study conducted at a single facility, making selection bias unavoidable. In the conference, it was determined that some cases judged unsuitable for triplet therapy included “truly frail” cases, and doublet regimens or other treatment options were selected. In other words, the cases analyzed were limited to those judged to be “likely manageable with triplet regimens.” In addition, in the supplementary review, 13 patients who were determined to be “frail” at the conference and assigned to a treatment plan involving doublet regimens or observation without treatment were included during the analysis period, accounting for 15% of all frail patients.

## 5. Conclusions

The conclusion from the present study is that, in frail MM patients able to undergo a triplet regimen, the anti-CD38 antibody-containing triplet regimen demonstrated an efficacy and safety equivalent to that in non-frail patients, suggesting that it may remain a useful treatment option for frail MM patients. Amidst the rapid evolution of MM treatments, for example, in CAR-T therapy, various bispecific antibody drugs, and quadruplet combination regimens, it is essential to carefully select treatment options for frail patients.

## Figures and Tables

**Table 2:** Summary of relative dose intensity.

	Frail (*n* = 71) (%)	Non-Frail (*n* = 79) (%)	All (*n* = 150) (%)	*p*-Value
Daratumumab RDI, mean (range)	99 (50–100)	100 (25–100)	100 (25–100)	0.21
Isatuximab RDI, mean (range)	100 (50–100)	92 (25–100)	96 (25–100)	0.71
Lenalidomide RDI, mean (range)	93 (18–245)	82 (31–225)	84 (18–245)	0.78
Pomalidomide RDI, mean (range)	86 (47–100)	82 (33–145)	84 (33–145)	0.82
Bortezomib RDI, mean (range)	92 (67–100)	100 (55–100)	100 (55–100)	0.06
Carfilzomib RDI, mean (range)	74 (50–100)	99 (58–100)	98 (50–100)	0.26
Dexamethasone RDI, mean (range)	87 (28–100)	97 (25–100)	92 (25–100)	0.09

RDI = relative dose intensity.

**Table 3:** Initial dose and reduction rate of therapeutic agents.

	Frail (*n* = 71)	Non-Frail (*n* = 79)	All (*n* = 150)	*p*-Value
Lenalidomide, initial dose (mg), mean (range)	15 (5–25)	10 (10–25)	10 (5–25)	0.49
Lenalidomide reduction rate, (%)	55	68	61	0.32
Pomalidomide, initial dose (mg), mean (range)	3.5 (2–4)	4 (2–4)	4 (2–4)	0.009
Pomalidomide, reduction rate, (%)	50	8.7	24	0.014

**Table 4:** Reasons for discontinuation of treatment.

	Frail (*n* = 71) (%)	Non-Frail (*n* = 79) (%)	All (*n* = 150) (%)	*p*-Value
Disease progression or lack of efficacy	36 (51)	49 (62)	85 (57)	0.45
AEs	6 (8.5)	12 (16)	18 (12)	
Other disease progression	2 (2.8)	1 (1.3)	3 (2)	
Others	5 (7)	3 (3.8)	8 (5.3)	

AEs = adverse events.

**Table 5:** Summary of adverse events.

	Frail (*n* = 71) (%)	Non-Frail (*n* = 79) (%)	All (*n* = 150) (%)	*p*-Value
Total AE	71 (100)	79 (100)	150 (100)	
Grade 1–2, *n* (%)	5 (7)	11 (14)	16 (11)	
Grade 3–4, *n* (%)	66 (93)	68 (86)	134 (89)	0.20
Hematological AE				
Anemia				0.06
Grade 1–2, *n* (%)	42 (59)	57 (72)	99 (66)	
Grade 3–4, *n* (%)	28 (39)	21 (27)	49 (33)	0.12
Neutropenia				0.36
Grade 1–2, *n* (%)	16 (23)	18 (23)	34 (23)	
Grade 3–4, *n* (%)	39 (55)	48 (61)	87 (58)	0.62
Lymphocytopenia				0.63
Grade 1–2, *n* (%)	15 (21)	19 (24)	34 (23)	
Grade 3–4, *n* (%)	54 (76)	54 (68)	108 (72)	0.85
Thrombocytopenia				0.78
Grade 1–2, *n* (%)	40 (56)	39 (49)	79 (53)	
Grade 3–4, *n* (%)	21 (30)	26 (33)	47 (31)	0.73
Non-hematological AE				
Any infection				0.29
Grade 1–2, *n* (%)	16 (23)	13 (16)	29 (19)	
Grade 3–4, *n* (%)	13 (18)	8 (10)	21 (14)	0.26
Pneumonia				0.47
Grade 1–2, *n* (%)	1 (1.4)	3 (3.7)	4 (2.7)	
Grade 3–4, *n* (%)	10 (14)	6 (7.6)	16 (11)	0.29
COVID-19				0.34
Grade 1–2, *n* (%)	4 (5.6)	1 (1.3)	7 (4.7)	
Grade 3–4, *n* (%)	2 (2.8)	0 (0)	2 (1.3)	0.50
CMV infection				0.19
Grade 1–2, *n* (%)	4 (5.6)	1 (1.3)	5 (3.3)	
Grade 3–4, *n* (%)	0 (0)	1 (1.3)	1 (0.6)	0.60
VZV infection				
Grade 1–2, *n* (%)	2 (2.8)	1 (1.3)	3 (2.0)	0.6
Grade 3–4, *n* (%)	0 (0)	0 (0)	0 (0)	
Sepsis				
Grade 1–2, *n* (%)	0 (0)	0 (0)	0 (0)	
Grade 3–4, *n* (%)	2 (2.8)	2 (2.5)	4 (2.7)	0.29
Rash				0.31
Grade 1–2, *n* (%)	4 (5.6)	2 (2.5)	6 (4.0)	
Grade 3–4, *n* (%)	1 (1.4)	0 (0)	1 (0.6)	1.0
Thromboembolism				
Grade 1–2, *n* (%)	3 (4.2)	2 (2.5)	5 (3.3)	0.67
Grade 3–4, *n* (%)	0 (0)	0 (0)	0 (0)	
Osteonecrosis of the jaw				1.0
Grade 1–2, *n* (%)	2 (2.8)	0 (0)	2 (1.3)	
Grade 3–4, *n* (%)	0 (0)	3 (3.8)	3 (2.0)	

CMV = cytomegalovirus; VZV = varicella zoster virus.

## Data Availability

The data presented in this study are available upon request from the corresponding author. The data are not publicly available due to privacy restrictions.
